# Isotropic non-contrast whole-heart lumen only coronary MRA using local re-inversion and 2D-SENSE at 3 Tesla

**DOI:** 10.1186/1532-429X-13-S1-O26

**Published:** 2011-02-02

**Authors:** Harsh K Agarwal, Jing Yu, Micheal Schär, Allison Hays, Robert G Weiss, Matthias Stuber

**Affiliations:** 1 Johns Hopkins University, Baltimore, MD, USA; 2 Philips Healthcare, Cleveland, OH, USA

## Introduction

Double-inversion recovery MRI with a cylindrical re-inversion prepulse has been presented for selective visualization of the coronaries [1]. Local re-inversion (LoReIn [1]) labels the blood in the ascending aorta and LV, and acquires images after a labeling delay providing coronary MRA(cMRA) of the blood flow with excellent suppression of myocardial signal. LoReIn was now implemented and tested for whole-heart cMRA [2] at high magnetic field strength.

## Purpose

To develop a whole-heart cMRA of the blood flow during a labeling delay with complete suppression of myocardial signal at 3T.

## Methods

Whole-heart cMRAs were obtained in four healthy volunteers during free breathing on a commercial Phillips Achieva 3T MR scanner using a 32-channel cardiac coil. A 3D gradient-echo sequence (TR/TE=3.3ms/1.09ms; α=20°; acquisition window=107.5ms; FOV=272mmx232mmx120mm; voxel size=1.3mmx1.3mmx1.3mm; SENSE=2 in foot-head direction; SPIR fat saturation) was acquired twice first with T2-preparation[2,3] and then with LoReIn preparation to generate blood-myocardium contrast. A 25mm radius 2D cylindrical shaped labeling pulse through the ascending aorta was applied after the R-wave and images were acquired after a labeling delay [4] to suppress the myocardial (T1=1200ms) signal at 3T. Imaging every other heart beat enabled a labeling delay of 550-650ms to capture the two peaks of pulsatile blood flow [5] and image acquisition during mid-diastole. Scan time(320 heart beats) was kept constant by changing the fold-over direction and using an additional SENSE factor of 2 in right-left direction. SNR of blood, blood-myocardium CNR, and vessel length were statistically compared using Student’s t-test for significance below p-value of 0.05.

## Results

**Figure 1:**
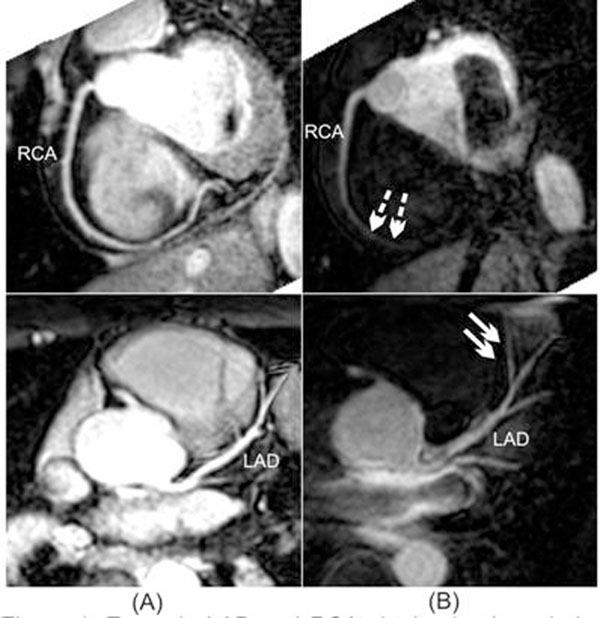
Example LAD and RCA obtained using whole-heart cMRA with (A) T2-prep and (B) proposed local re-inversion pre-pulse. Solid arrow marked the suppression of myocardium with local re-inversion pulse and dotter arrow marks the length of blood flow in RCA during the prolonged labeling delay at 3T.

All scans were successfully completed with both techniques. An example reformatted RCA and LAD in Figure 1 shows the suppression of myocardium with LoReIn and the extent of blood flow into the RCA during the labeling delay. The quantitative results are listed in Table 1. Visible vessel length of LAD and LCx is similar between the two scans, however, a longer RCA is visualized for T2-prepared scan which is independent of the in-flow of blood. SNR of blood is similar for both techniques, however, the CNR between blood and myocardium is significantly higher with LoReIn.

**Table 1:** Quantitative comparison of cMRA obtained using T2-Prepared and local re-inversion pre-pulse

	Whole-heart T2-Prepared cMRA	Whole-heart local re-inversion prepared cMRA
SNR blood	116±51	121±41
CNR blood and myocardium	63±35	121±41*
RCA length (mm)	116.9±13.8	55.6±12.2*
LCX length (mm)	57.1±12.1	53.6±7.0
LAD length (mm)	57.0±20.1	50.7±11.2

*p<0.05 using Student’s t-test

## Conclusions

2D-SENSE accelerated isotropic whole-heart cMRA of the blood flow during a labeling delay with suppressed fat and myocardium has been successfully developed and applied at 3T for visualizing continuous segments of major coronary arteries.
